# British Association for the Advancement of Science

**Published:** 1838-10

**Authors:** 


### BRITISH ASSOCIATION FOR THE ADVANCEMENT OF SCIENCE.

The eighth meeting of this admirable Society took place this year at Newcastle, in the week beginning Monday, August 20th, and was most fully attended. Our limits permit us only to notice the proceedings in the Medical Section; and we thankfully acknowledge that we are indebted for the account of these, to The Athenæum, a publication which has always given admirable reports of the meetings, and which deservedly now stands at the head of the weekly literary journals of this country.

### Section E.—MEDICAL SCIENCE.

*President.*—T. E. Headlam, M.D.
*Vice Presidents.*—Prof. Wm. Clark, M.D., John Yelloly, M.D., Mr. John Fife.
*Secretaries.*—Mr. T. M. Greenhow, J. A. Vose, M.D.
*Committee.*—P. M. Roget, M.D., A. B. Granville, M.D., R. S. Sargent, M.D., B. T. Evanson, M.D., C. J. B. Williams, M.D., J. C. Prichard, M.D., Mr. John Baird, W. Lynch, M.D., N. T. Smith, M.D., Mr. William Fife, R. Reed, M.D., Charles Wightman, M.D., J. Carson, jun. M.D., Prof. Owen, J. Reid, M.D., R. D. Thomson, M.D., Jos. Brown, M.D., Mr. Wm. Morrison.

(Monday, *Aug.* 20.)

A paper was read by Mr. T. M. Greenhow, "On the Beneficial Action of Mercury rapidly introduced in certain cases of Neuralgia."

After some general remarks on mercury as an important therapeutic agent, he stated the following conclusions, as generally derivable from its usual effects: 1st. In secondary syphilis, when slowly introduced into the system, much valuable time was frequently lost, as in iritis. 2d. That the injurious effects of it, which were sometimes observed to follow its operation in syphilis, did not follow when administered for liver disease. 3d. In syphilis, if not quickly beneficial, it proved detrimental, being either a "bane or antidote." 4th. That the mode of administration frequently decided which operation followed; and 5th. That in iritis and amaurosis, the beneficial effects were proportioned to the rapidity of its introduction into the system. Two cases were then detailed, in which the rapid introduction of mercury into the system proved completely successful, in removing severe and long-continued neuralgia,—the first occurring after a severe attack of hemiplegia, which was removed by ordinary treatment. The neuralgia recurred once after removal from the lower extremity in another situation, in the shoulder and arm, and was again relieved, and permanently, by the rapid action of calomel and opium. The second case of severe neuralgia occurred in a lady, who previously had given birth to still-born children. The violence and long continuance of the disease resisted all the usual external and internal remedies, and had shattered both mental and bodily health. The disease completely disappeared on the rapid introduction of mercury into the system, though it had previously resisted the slow action of the same remedy. Other painful afflictions were adduced, in proof of the efficacy of this mode of treatment, that could not be suspected of having any connexion with a syphilitic taint in the constitution.

Dr. Granville enquired as to the degree of salivation induced.—Mr. Greenhow replied, that a decided affection of the gums with slightly increased action of the salivary glands was enough, without profuse salivation.—Dr. Brown confirmed the observations contained in the paper, by cases in which mercurial action removed severe neuralgia, not connected with inflammation or syphilis.

Mr. R. M. Glover read a paper, "On the Functions of the Rete Mucosum and Pigmentum Nigrum in the dark races, and particularly in the Negro; with Observations on a paper on the same subject by Sir Everard Home."

The paper commenced by stating, that the degree of development of the rete mucosum and its pigment, determined the power of resisting the excessive heat of the sun in tropical climates, as evinced by the Negro, (the type, in this respect, of the dark races) the European, and the Albino. The *modus operandi* must be discovered by an attention to both the physical and vital properties of this peculiar organization. The doctrine at present taught on the subject is, that the black skin absorbs more heat, but that the cutis vera of the negro is not so liable to inflammation from a high temperature, as that of a European from a lower temperature; and, as the radiation of caloric from black must be greater than from white skins, the possessor of the former must cool more readily, and enjoy greater alternations of heat and cold. The former part of this doctrine is founded on the experiments and deductions of Sir Everard Home, as detailed in his paper in the Philosophical Transactions. A number of experiments detailed in the paper on the vesicatory powers of differently coloured substances, under the concentrated rays of the sun, contradicted the deductions of Sir E. Home; and hence arose the necessity of looking to the vital properties of the skin of the negro, and the mode in which it is likely to be affected by the radiating and absorbing power of the pigment with which he is provided. Blumenbach and Winterbottom state, that the negro perspires more readily and freely than the European; and Davy says, "In the inhabitants of the tropics, the exhalent arteries of the skin seem unusually expanded, and the whole apparatus peculiar to this secretion unusually developed; and I believe that the blood itself is less viscid, more fluid, and flows more readily through the vessels, so as to promote perspiration, and by that means contributing to the cooling of the surface. And being cooled itself, it contributes again, when it flows back upon the heart, to the reduction of the temperature of the internal parts." Were the inhabitant of the tropics not possessed of this peculiar organization, his system could not respond to the stimulus of heat, by a determination of fluid towards the surface. Doubtless, the excessive absorption of heat by his skin, is useful in promoting this effect; but in the system qualified to respond to the stimulus of heat, and not in the organization of the skin alone, must an explanation be sought of the capability of the negro to withstand the heat of tropical regions.

In the next paper, Dr. John Reid gave an account of "An Experimental Investigation of the Functions of the Eighth Pair of Nerves."

This communication contained some of the principal results which Dr. Reid has obtained in prosecuting this enquiry since the last meeting of the Association. Dr. Reid chiefly confined himself to the Pulmonary and Gastric branches of the Nervus Vagus. In a great number of experiments upon dogs, in which the nervi vagi and recurrents were divided, Dr. Reid satisfied himself that, when a sufficient quantity of air reached the lungs, the respirations were at first performed with ease. The only immediate and constant effect observed, was a great diminution in the number of the respiratory movements, which at the same time became slower and more heaving. When the number of respirations were from sixteen to twenty in a minute, they, in general, instantly fell to from six to eight. In one animal, in which they were from twenty-four to twenty-eight, they immediately fell to fourteen; and, in another animal, in which they were twenty-four, they fell to eight. At a longer or shorter period after section of the nerves, the inspirations became more prolonged and heaving, while the expirations continued to be comparatively short and rapid, and attended by a sound caused by the sudden expulsion of the air. After a time, the inspirations become more heaving and prolonged, the blood is less perfectly arterialized in the lungs, and the arteries circulate blood gradually approaching the venous character; the animal becomes dull and stupid, and dies asphyxiated. In tracing the morbid changes upon the lungs, which follow section of the vagi, Dr. Reid has satisfied himself, from the dissection of animals killed at various periods after section of the vagi, that the blood-vessels of the lungs become gradually loaded with blood, and that this, in a few cases, is the only change which can be observed after death. More frequently, however, this congestion of the lungs gives rise to effusion of serum, which, in the bronchial tubes, is rendered frothy by the passage of air through it; to condensation of portions of the lung without any apparent effusion of lymph; and more rarely to true pneumonia. The respiratory murmur is little, if at all, changed for a short time, but it afterwards becomes bronchial; and when the effusion of serum takes place, the true *râle crepitant* may be heard. It appears, however, that these morbid changes in the lungs do not necessarily take place after section of both vagi and recurrents. In a dog, which was killed in the presence of Dr. Alison, twelve days after section of the vagi and recurrents, and which had apparently recovered from the effects of the operation, the lungs were found perfectly healthy, though the cut ends of both vagi were rather more than one inch distant from each other. Dr. Reid believes that the diminished frequency of the respiratory movements is sufficient to explain all the morbid changes in the lungs, which result from section of the nervi vagi. He related several experiments to prove, that the respiratory movements are not arrested by division of the vagi and recurrents after removal of the cerebrum and cerebellum. In these experiments, it was, however, observed, that though section of the vagi did not arrest the respiratory muscular movements, they were much diminished in frequency. While these experiments illustrate the great importance of the par vagum as exciters of respiration, they also prove that there are other nerves which can transmit those impressions to the medulla oblongata, which excite the respiratory movements; and Dr. Reid adduced some facts to show, that one of the most important of these was the larger root of the fifth pair distributed upon the face. Dr. Reid then related four experiments, which he considered to afford satisfactory evidences of the continuance of digestion after section of both vagi, with loss of substance. In three of these, the lacteals and thoracic duct were found full of chyle. From these experiments, he concluded, that, though by division of the vagi a deleterious influence is propagated downward to the stomach, disturbing the functions of the digestive organs; yet, if the animal live long enough, this goes off, and digestion proceeds as before. He related some experiments to prove, that alcohol, opium, and prussic acid exert their deleterious effects as rapidly when injected into the stomach after division of the vagi, as when these nerves are left entire. He then detailed the result of five comparative experiments upon the effects of fatal doses of arsenic upon the watery and mucous secretions from the inner surface of the stomach and intestines. These results were completely at variance with the statement of Sir B. Brodie, that section of the vagi arrests the usual mucous and watery secretions of the stomach and intestines observed after poisoning with arsenic. He stated, that he had of late carefully watched the effects of division of the sympathetic nerve on the eye, and that he had satisfied himself that the contracted state of the pupil, and the partially-closed eyelids, which accompany the inflammation of the conjunctiva, consequent upon section of the sympathetic, take place previously to the inflammation of the conjunctiva, and are independent of it.

Prof. Owen requested to know, if albumen were discovered in the thoracic duct after the division of the vagi nerves. Dr. Reid replied, that no chemical analysis was made of the contents, but true digestion was inferred by him from the return of health and activity to the animal operated on.—Dr. Golding Bird pressed the same objection as Professor Owen, and wished that an experiment on the subject should be followed up by such a chemical investigation as would be decisive.

(Tuesday, *Aug.* 21.)

The Section did not meet until half-past eleven o'clock, as the greater number of members had gone, by invitation, to be present at the operations performed in the infirmary. The first paper was read by Mr. Torbock on Uterine Hæmorrhage, the object of which was to introduce a new mode of applying pressure in certain forms of the disease. A few cases were adduced in proof of its efficiency.

A conversation ensued, in which the danger of relying on any *merely* mechanical treatment, to the exclusion of fundamental principles previously established, was pointed out by Drs. Granville, Lynch, and Knott.

Dr. Robert D. Thomson then read a paper, by Mr. N. Farr, "On the Law of Recovery and Mortality in Cholera Spasmodica."

Dr. Thomson introduced his subject by making some general remarks upon the importance of applying the precise language of mathematical science to all branches of natural knowledge; and, after apologising for bringing mathematical formulæ before medical men, stated that the only mode of placing medicine on a level with the sciences which are of a demonstrable, and therefore irresistible nature, will be by establishing it on a mathematical basis—for until we can calculate with absolute precision on the natural laws of diseases, and on the action of remedies, we can be only said to labour in our vocation like the ancient mariners, who could never venture beyond the confines of the ocean without suffering the most disastrous consequences. Dr. Thomson had been induced to bring the present subject before the Medical Section at the request of the able author, whose absence he regretted, for the purpose of showing that the law which regulated the disease so well known under the name of Indian cholera was as precise as any of those which guided the heavenly bodies in their courses. It was a curious fact that the state of medicine was so far in arrear in this country, that, although numerous cholera hospitals had been established in this country during the prevalence of that disease, not one of their conductors had paid any attention to the proper mode of registering the features of the disease, so as to render them available for drawing conclusions of any value; and that the only place which had hitherto afforded the proper data for this purpose was Rome, a city to which many persons were ignorantly inclined to deny any credit in a scientific point of view. The subsequent conclusions were deduced from 9372 registered cases, in 1837, published by the Roman Board of Health in their report, for which the author was indebted to Sir James Clark. It affords a striking proof of the importance of Boards of Health, and of the great superiority of the Papal provisions for the health of the inhabitants, and formed a striking contrast to the laws of England. It would be highly desirable, in the absence of such provisions by government, that physicians of hospitals should decide upon a uniform method of registering the principal features of diseases. The principal points to attend to were the *day of the disease*, or day of attack: second, the *day of recovery*, distinguishing males from females; and third, the *day of death*. From data of this kind the extensive tables presented to the meeting were deduced. One of these tables exhibited the numbers dying and recovering on each day after attack, and contained calculations from theoretical considerations, which closely approached the amounts derived from facts. The following table will show this more clearly:—

Out of one hundred cases constantly sick:

| | Deaths. | | Recoveries. | |
|---|---|---|---|---|
| | Observed. | Calculated. | Observed. | Calculated. |
| 5th day... .. | 5.471 | 5.650 | 6.747 | 6.747 |
| 6th............ | 5.684 | 5.056 | 8.295 | 7.929 |
| 7th............ | 4.500 | 4.523 | 9.219 | 9.317 |

The following table expresses the probability of recovery and death during the first ten days after attack:

| Days. | Probability of Recovery. | Probability of Death. |
|---|---|---|
| 0 | ...... .422 | ...... .578 |
| 1 | ..... .542 | ...... .458 |
| 2 | ...... .668 | ...... .332 |
| 3 | ...... .729 | ...... .271 |
| 4 | ..... .763 | ...... .237 |
| 5 | ...... .791 | ...... .209 |
| 6 | ...... .821 | ...... .179 |
| 7 | ..... .843 | ...... .157 |
| 8 | ... .. .862 | ...... .138 |
| 9 | ...... .873 | ...... .127 |
| 10 | ... .. .883 | ...... .115 |

From the tables may be deduced the following problems: viz. 1st, the mean duration of the disease; 2d, the mean future duration of the disease at any period; 3d, the probability of dying at any period of the disease.

Dr. Granville deprecated the attempts to introduce laws mathematically drawn or expressed, into the immediate subjects of medical enquiry such as the present. The laws derivable from physical enquiries, such as astronomy, had their value from the known laws of gravitation, but no similar fundamental law was known in physiology; and the innumerable modifying circumstances, such as differences of age, sex, treatment, and diversity of constitution, would interminably disturb any deductions thus made on the questions of disease.—Dr. Thomson, in answer, referred to the close coincidence of the law (expressed by a mathematical curve) and the observations recorded, and explained some points in the structure of the tables.—Dr. Sargent differed from Dr. Granville as to the impracticability of ever arriving at any approximation to such a law as that now exhibited. It was impossible to view the tables before them, even cursorily, to compare them with the manifest deduction as to regular gradations, and with his own observations on the gradual diminution of the mortality of the dreadful epidemic in several invasions, without coming to the conclusion, that the coincidence or approximation of the observations and calculations must have a foundation in nature. As to the modifying circumstances mentioned, these were difficulties, no doubt, not to be overlooked: and did not disturbing elements and difficulties occur in physical investigations?

Mr. Joseph Blake then read a paper "On the action of various Substances on the Animal Economy, when injected into the Veins," in which were detailed a number of experiments with various substances, and their effect on the vascular system, measured by an ingenious instrument, which the author denominated a Hæmadynameter, formed by a glass tube, having both extremities proceeding from an angle,—one being attached to a scale, measuring the height to which a column of mercury was raised by the action of the current of blood in the artery, into which the other extremity was introduced. The substances introduced, in solution, into the veins were divided into three classes, from their effects. In the first, were those which produced death, by directly acting on the contractility of the heart, amongst which were nitrate of potassa, arseniate of potassa, sub-carbonate of soda, biniodide of arsenic, oxalic acid, and solution of galls; all these acted locally on the heart, and agreed in effecting a change in the colour of the blood, turning it black, probably by forming definite combinations with its constituents. A remarkable difference was observable from the effects of the same substances when absorbed from the stomach. In the second class, were those substances which acted directly on the nervous system; such were strychnia, hydrocyanic acid, and conia. And in the third, were those producing death by affecting the capillary circulation; such were tobacco, euphorbium, and digitalis. The last two classes of substances did not produce any change on the composition of the blood. Several other substances were experimented with, not falling under the above classes, such as morphia and cantharides, the effects of which were the same,—and nitric acid: when the latter was injected into the vein, the column of mercury in the instrument fell from seven inches to one; and after death, the right side of the heart was distended with solid blood.

Dr. Lynch and Mr. Torbock corroborated the observations made in the first class of substances employed, having observed the same changes in the blood produced by acids under different circumstances.

Dr. Barnes then read a paper, containing a report of two cases of well-marked abscess of the lungs from acute inflammation.

(Wednesday, *Aug.* 22.)

Dr. Yelloly (the Chairman) laid before the Section, a model of an improved acoustic instrument, for the purpose of assisting in cases of partial deafness. He alluded to the very defective nature of our present instruments, both as to utility and convenience, and the importance of having some experimental researches made on the subject; and he proposed, that the Section recommend to the General Committee the appointment of a committee to investigate the subject experimentally, and to make a report on the subject, at the next meeting of the Association. Dr. Granville seconded the motion, and proposed that Dr. Yelloly and Dr. Arnott should be named as the Committee.

Professor Owen then read a paper "On the Structure of Teeth, and the resemblance of Ivory to Bone, as illustrated by microscopical examination of the teeth of man, and of various existing and extinct animals."

Mr. Owen commenced by showing, that he had availed himself of the advantage afforded by the British Association—viz., that in the communications brought before a sectional committee, a fuller and more detailed retrospect of the progressive steps which have led to any remarkable discovery, is not only permissible, but peculiarly congenial to its general views and objects; and he therefore entered into a full detail of the recent investigations, especially those of Purkinje, Müller, and Retzius, on the intimate structure of the teeth, and particularly dwelt on the discoveries of the latter author, as regarded the structure of the human tooth. After describing the mode of arrangement of the particles of the earthy salts, which characterizes true bone, Professor Owen proceeded to state, that until a very recent period the analogy of tooth to bone was supposed to extend no farther than related to the chemical composition of the hardening material, while the arrangement of this earthy constituent, as well as its mode of deposition during the growth of the entire tooth, were considered to be wholly different from that of bone, and to agree with the mode of growth of hair, and other so-called extra-vascular parts, with which the teeth undoubtedly closely correspond in the general vital properties. He observed, that the supposed proofs of the laminated structure of teeth, derived from the appearances presented by the teeth of growing animals, fed alternately with madder and ordinary food, and by those which often occur during the progress of decomposition of certain teeth, which are then resolved into a series of concentric or superimposed laminæ, were equally applicable to true bone, and were quite unavailable in illustrating the point under consideration: and that the appearances presented by the superficies of vertical sections of teeth, viewed with the naked eye or a low magnifying power, were due, not to the intervals of separate and superimposed lamellæ, but to the different refractions of light, caused by the undulation of a series of parallel tubes proceeding in a contrary direction to the supposed lamellæ. This apparent lamellated structure, however, is not constant, nor equally plain in different teeth; on the contrary, the fractured surface, or the polished section of the human and many other teeth, presents a silky or iridescent lustre, which has attracted the attention of several anatomists. Professor Owen observed, that Malpighi, in whose works may be detected the germs of almost all the great anatomical truths, which have subsequently been matured and established, conceived that the teeth were composed of minute fibres reticularly interwoven; and Leewenhoek in

1683, had discovered, that the apparent fibres of tooth were, in reality, minute tubes. The tubular structure of ivory was rediscovered by Purkinje, in 1835, who also added several interesting facts regarding the structure of the enamel, and more especially determined the nature of that distinct layer of substance, which surrounds the fang in the simple teeth of man and carnivorous animals. The interesting experiments of Professor Müller, on the nature and contents of the *dental tubuli* were then noticed; and, lastly, a condensed analysis was given of the laborious and accurate microscopical observations of Professor Retzius, as related in the original Swedish memoir of that author, on the structure of the teeth. Besides confirming the fact, that the ivory or bony constituent of a tooth consists of the fundamental *zahn-substanz* of Purkinje, minute tubes lodged in a transparent medium, disposed in a radiated arrangement in lines, proceeding in a direction perpendicular to the superficies of the tooth, Professor Retzius has more particularly observed, and described the dichotomous branching of the primary tubes; the minuter ramuli sent off throughout the course of the main tubes into the clear interspaces; the calcigerous cells with which those fine branches communicate, and the terminal ramifications of the tubuli; their anastomoses with each other, and with calcigerous cells at the superficies of the ivory, or bony part of the tooth. Professor Owen also discussed the opinion advanced by Professor Retzius, as to the function of this elaborate contexture of branched and anastomosing tubes and cells, in conveying, by capillary attraction, a slow current of nutritive or preservative fluid, through the entire substance of the tooth; which fluid might be derived either from the superficies of the pulp in the internal cavity of the tooth, or from the corpuscles or cells of the external layer of cortical substance or *cæmentum*,—with the tubes radiating from which corpuscles, the fine terminal tubes of the ivory anastomose. Professor Owen concluded the critical portion of his communication, by explaining the views entertained by Professor Retzius on the analogy subsisting between tooth and bone, which analogy he then proceeded to illustrate by his own observations on the structure of recent and fossil teeth. [For the descriptions given by Professor Owen of these teeth we cannot find room.]

In the summary of the series of observations which Professor Owen detailed, he observed, that in the human tooth, and similarly highly organized teeth, the analogy of ivory to bone, as to texture, was only seen in the existence and intercommunication of the minute calcigerous tubes and cells; but that there was no trace of medullary or Haversian canals with their characteristic concentric laminæ, unless the entire tooth were regarded as analogous to a single enlarged Haversian canal; the cavity of the simple pulp representing the medullary cavity of the canal; while the tubes, with the appearance of laminæ occasioned by their undulations, were equivalent to the concentric lamellæ and the calcigerous tubes, which, in bone, traverse these lamellæ, and radiate from the Haversian canal. In the teeth of many of the lower animals, however, and especially that of the extinct *Acrodus*, amongst the cartilaginous fishes, the resemblance of the dental tissue to bone was extended to the existence of the characteristic Haversian canals in great numbers. The presence of these canals was explained by the progress of the development of these bone-like teeth, as observed by Prof. Owen in recent cartilaginous fishes. The large pulp, at the commencement of the formation of the tooth, had exercised its ordinary function in the secretion of a close-set series of calcigerous tubes, having a general direction perpendicular to the surface of the tooth, and closely resembling true ivory. The pulp, then, instead of continuing to form similar tubular ivory, by adding to the extremities of the previously formed tubes, become subdivided, or broken up into numerous processes, to which those forming the three fangs of a human grinder are analogous; but each process here becomes the centre of an active formation of similar branched tubes, radiating in all directions from that centre, and anastomosing, by their peripheral branches, with those from contiguous centres, or communicating with interposed calcigerous cells: the cavities containing the above subdivisions of the pulp, like the Haversian canals, containing the processes of medulla in true bone, have had their area diminished in like manner by the successive formation of a series of concentric lamellæ; traversed, as in true bone, by radiating and minutely ramified calcigerous tubes, communicating with each other and with the minute cells in the interspaces.

The resemblance of the pulp canals of the teeth of *Acrodus* and of the medullary canals of bone, is further exemplified in the existence of lateral communications in teeth; and, in function as well as structure, they might be regarded as being identical. With reference to the application of the tubular structure of the teeth to the explanation of their pathology, nothing has hitherto been attempted. Prof. Owen observed, that it was a new and fertile field, which would doubtless be replete with interesting results, and might suggest some good practical improvements in dental surgery. Ordinary decay of the teeth commenced, in the majority of instances, immediately beneath the enamel, in the fine ramifications of the peripheral extremities of the tubes, and proceeded in the direction of the main tubes, and, consequently, by the most direct route to the cavity of the pulp. The decayed substance, in some instances, retains the characteristic tubular structure, which is also observable in the animal basis of healthy teeth after the artificial removal of the earthy salts. The soft condition of the decayed portion of a tooth is well known to all dentists: it depends upon the removal of the earthy salts from the containing tubes and cells, in which process the decay of teeth essentially consists. The main object of the dentist seems, therefore, to be, to detect those appearances in the enamel which indicate the commencement of decay; to break away the enamel, whose natural adhesion to the ivory will be found more or less dissolved at the decayed part; to remove the softened portion of the ivory, and fill up the cavity with gold or other substance. Experience proves what theory cannot account for,—viz. that the progress of the decay is sometimes thus permanently arrested. The calcareous salts are in such cases, as it were, poured out from the extremities of the tubes divided in the operation, and a thin dense layer intervenes between the exposed surface of the ivory and the stopping.

In conclusion, Professor Owen passed in general review over the structures which he had described in detail, and remarked, that through the endless diversity which the teeth of different animals presented, the general law of the tubular structure could be unequivocally traced; and that the general tendency of the modifications observable in descending from man to the lower classes of vertebrate animals, was a nearer and nearer approximation of the substance of the tooth to that of ordinary bone.

Numerous sections of the teeth described, prepared for microscopic examination, were exhibited to the Section.

Dr. Reid then gave a brief notice of his researches "On the Quantity of Air required for Respiration." He pointed out imperfections in previous experiments, particularly that consisting in the small number of individuals experimented on. A great difficulty existed in attempting accurate conclusions, from the diversity of constitutional temperaments, different states of humidity of the atmosphere, the state of insensible perspiration, and also from the admixture of small quantities of foreign gases; in one instance, the admixture of $\frac{1}{3000}$ part sulphuretted hydrogen, was enough to "knock up" a whole room, producing very serious effects. The degree of light was also an important element. Dr. R. stated, that, in recovering from the effect of various oppressive atmospheres, to which he was necessarily at times subjected, and some of which contained ten per cent. of carbonic acid, he always recovered more quickly, and felt in a short time more thoroughly refreshed, if he was exposed, not only to a fresh and free atmosphere, but also to a brilliant light. He added, that at St. Petersburgh, he was informed by Sir James Wylie, that the cases of disease on the dark side of an extensive barrack, were in the proportion of three to one, to those on the side exposed to strong light, and this uniformly so for many years. Dr. Reid explained the mode he had adopted to ventilate the House of Commons, which he illustrated by diagrams, and demonstrated by the exhibition of a glazed model of the House. The current of fresh air could be introduced either from below or from above, diffused uniformly, and not by violent draughts, but as it were insensibly, and was under the most exact control as to quantity. The air, when used for the purposes of respiration, and combustion, was conveyed away in an opposite direction to that in which it had been introduced. In answer to a question, Dr. Reid said, that he had taken no account of the products of the combustion by which the heat and light were produced, as these products should be omitted in all calculations on the subject. They, if possible, should be carried off so as not to interfere with the immediate supply to each individual. For the purpose of raising the temperature, hot water was used in iron tubes, not raised above 150°. Dr. Reid also stated, in answer to other questions, that he had not made any particular observations on the modifying influence of different articles of clothing; but he believed they did modify considerably the question; those being preferable that were of a very porous nature, allowing an *insensible* application of the atmosphere to the cutaneous surface.

Dr. Inglis read a paper containing " Remarks on the Skull of Eugene Aram."

Proofs of the identity of the skull were first adduced. The head was removed from the body, which, according to the sentence, was hung in chains at Knaresborough, by Dr. Hutchinson, of that town, whose widow married a gentleman still living, from whom it passed into the hands of the Rev. Mr. Dalton, from whom Dr. Inglis received it. Mr. Dalton, it appears, sent it to Spurzheim, who mistook it for a female skull. The paper stated that the development of the mental faculties, as indicated by the skull, agreed in a remarkable manner with the character of Aram as recorded. Strictures on the nature and amount of the evidence by which he was condemned were brought forward; and these strictures were enforced by the probable character of Aram, as indicated by the skull. A long and desultory conversation ensued, in which the evidence of the identity of the skull was denied by some members, and acknowledged by others; and the importance and truth of phrenology were alternately asserted and denounced as chimerical and absurd.

(Thursday, *Aug.* 23.)

Previously to the reading of any papers, three recommendations to the general committee passed the Section:—1st. That a communication should take place with members of the Medico-Chirurgical Society of London, in order that papers read at this Section might be occasionally published in their Transactions. 2d. That application should be made for a grant of 200*l.* from the funds of the Association, for the purpose of bringing over to this country, and retaining here for one year, Alexis, mentioned by Dr. Beaumont, in his work on Digestion, for the purpose of making physiological and chemical researches on the subject of digestion, in connexion with the Chemical Section.* The committee proposed for the investigation were, Drs. Thomson, Prout, and Graham, and Professor Owen. 3d. For the appointment of a committee to communicate with previously appointed committees, for the purpose of furnishing reports on particular subjects for which pecuniary grants were given. This step was considered necessary, from the fact that no communication had been received from some of those committees.

Dr. Granville then laid before the section an improved Stethoscope, the advantages of which were the facility of examining the patient without the necessity of his rising, and also of avoiding the necessity of having the practitioner's head immediately parallel to the part examined: these advantages were effected by the addition of a half ball-and-socket joint attached to the ear-piece, which of course became moveable to a greater or less angle with the cylinder, as circumstances might require.

Dr. Rees read a paper "on the Chemical Nature of the Liquor Amnii." The principal points established were the specific gravity being found lower than the previous statements, being only 1.007, as an average of different results; that the fatty matter discovered in it was different from the fatty matter of the blood; and that urea was discovered in every specimen examined.

Dr. Bird observed, that this last fact tended to support the generally received opinion of German physiologists,—namely, that the liquor amnii was a secretion from the fœtal kidneys.

Mr. Baird detailed a case of successful excision of the elbow-joint. The patient was presented to the Section, and considerable motion was shown to exist in the joint; so much so as to enable him to pursue his ordinary occupation in a glass manufactory.

Dr. R. D. Thomson read a paper "on the Modus Operandi of Nitrate of Silver as a Caustic and Therapeutic Agent."

It was stated that light had no power whatever in decomposing nitrate of silver. In every instance in which this apparently occurred, it had been previously rolled in

* This was proposed by us in our last Number, p. 181.

paper, which afforded an opportunity for it to combine with organic matter, and thus produce the effect upon which the general opinion was founded. A specimen of pure nitrate of silver, placed in a tube hermetically sealed, was exposed on the top of a house for a considerable period, without the slightest discoloration. The specimen was exhibited. To illustrate the action of nitrate of silver when applied to the skin or a diseased surface, Dr. T. added a solution of this substance to a solution of white of egg, or albumen: he found that two distinct compounds resulted, one soluble, the other insoluble in water. He contended that these compounds were formed when it was applied to an organized surface, and that, they being removed, the caustic effect was thus produced; and that, in the internal use of this substance, the doses which could be administered in safety were proportioned to the quantity of albuminous matter contained in the stomach; and that, in the event of a deficiency, the coats of the stomach would be acted upon in the same manner, and death ensue; but that it was impossible for the substance unchanged to become mixed with the blood, and deposited on the surface, as was generally believed.

Mr. Greenhow read a brief memoir on Fractures, for the purpose of introducing to the Section a model of a new sling fracture-bed, applicable to every fracture in the lower extremity, but peculiarly adapted to the treatment of compound fractures of the femur. The following advantages were attainable by the present apparatus: 1st, ease of position; 2d, easy and gradual extension by means of a screw at the termination, beyond the heel of the patient, whose action was connected with the ankle-joint and instep; 3d, facility of examining and dressing the limb in cases of compound fracture, without disturbing the fractured ends; 4th, the freedom of slight motion, enjoyed in such a way as to be of no injury to the process of reparation. Mr. Greenhow detailed some interesting cases treated with this apparatus, demonstrating its peculiar advantages.

(Friday, *Aug.* 25.)

Dr. Bowring was introduced to the Section, for the purpose of communicating some observations on Plague and Quarantine, made during his residence in the East.

Dr. Bowring apologized for coming before the Section, he not being a medical man, but having travelled in the East for the purpose of observation, in reference to our commercial relations, his attention had been naturally directed to the subjects of plague and quarantine; subjects, the importance of which could hardly be overrated, many millions being annually lost to this country from quarantine regulations. The results of his observation had produced a strong conviction of the non-contagion of plague; and he thought it right, therefore, to lay before this Section a few remarks on the subject. He alluded to the very secondary character of the facts on which the prevalent opinions were founded. Some were so absurd as not to be worthy of the slightest attention: such as plague being introduced at Leghorn by the unrolling of a mummy that had been buried for 2000 years,—at Constantinople, by the wing of a bird having touched a kite which a boy was flying from a house-top,—from a cat having been seen to jump into a basket in which were some clothes, from which the disease was subsequently caught. Dr. Bowring said, that physicians residing in the East were rapidly changing their opinions on the subject; but they were prevented, in many instances, from freely expressing them, by the interested Boards of Health, who neither liked to part with their extensive power (even of life and death), or with their salaries. Clot Bey was a decided anti-contagionist, and that after an experience of 8,000 or 10,000 cases. Dr. Bowring mentioned many cases where facts were distorted, or invented, to account for cases of plague from contagion; and in one remarkable instance, where the misrepresentations were exposed, it was denied that the case was plague at all, because they could not maintain their assertion of contact having taken place. The Mussulmans are by their religion non-contagionists, and Dr. Bowring hoped they might never become otherwise, as the aggravation of the calamity would be tenfold if they did. The opinion as to the contagious nature of the disease prevailed principally amongst the Levantines and Franks; but every other superstition was as readily believed by them. He had collected the most solemnly attested evidence of the appearance of peris and genii, and of the intermarriages of the former with mortals: he had collected more strongly attested *facts* on these subjects than on the contagious nature of plague. Dr. Bowring asserted that, from innumerable instances, quarantine appeared to give no security; and was of opinion that these establishments were mere political engines, of great power and convenience in a despotic country. In the lazarettos, the whole correspondence of the East was read. The Russians had a most perfect system of quarantine, yet the plague got into Odessa. In 1831, quarantine and lazaretto establishments were introduced into Egypt, under the superintendence of the consuls; yet the plague got into Egypt. In Jaffa it broke out in the house of the Russian consul spontaneously; and in Jerusalem in a convent, with which there could have been no communication. In the lazarettoes, a little disease was made a great deal of: in one instance, a greater number died from dysentery than from plague. Lazarettos, he contended, rather increased than diminished the evil. If a strict separation could ensure safety, the pacha's harem would escape; yet, in 1835, seven died there of plague. It appeared at one time in Old Cairo, and not in New, and *vice vers* , although there was constant communication: the same was true as to Cairo and Alexandria. The disease never penetrated Nubia, though constantly on the borders, and frequent intercourse taking place. In Cairo, on one occasion, 400 or 500 houses, whose inhabitants had all perished, were subsequently opened, the linen and clothes in them sold in the market-place, without any cases of plague resulting. Clot Bey had again and again inoculated himself without producing the disease. Dr. Boulard wore the clothes of a patient, who died of the disease, for twenty-four hours, without catching it. The following information was communicated to Dr. Bowring, by a physician of long experience, in answer to a series of direct queries, viz.: that it is indigenous in Egypt, never entirely absent, never imported, that it frequently occurs spontaneously, that cordons afford no security, that contact very frequently did not produce it, and that the most cautious frequently suffered from it, that free ventilation was effective in checking the disease, that it was not produced by linen which had been exposed to the infection, and that when a number of persons exposed to its influence removed from the spot, the mortality became much diminished. Dr. Bowring concluded by expressing his own strong conviction on the subject; but he had no object but to promote the discovery of truth, which could only be done by patient and serious enquiry, and by evidence of a primary character.

Dr. Lynch begged leave to propose a resolution, to the effect, that Dr. Bowring's paper should be published.—Mr. Greenhow could not but remark on the strong analogy, which existed between the statements of the learned gentleman on plague and lazarettoes, and the events which occurred in this district relative to cholera and cholera hospitals. He also requested Dr. Bowring to furnish a copy of his observations to the Secretaries.—Dr. Granville opposed the motion of Dr. Lynch. The communication he contended was not strictly medical; it was political, not pathological: he must oppose too the deductions of Dr. Bowring; he was not indeed present at the commencement of his address, but that was the less necessary, as he had heard the whole subject in detail, on board the *Ocean* steam-packet. It was not by declaiming on the superstition of the Levantines, or by the eloquent introduction of Peris, Genii, and Vampires, that a strictly pathological question could be decided, nor by false inferences from bad Boards of Health.—The Chairman interrupted Dr. Granville, by protesting against this line of personal attack, and allusion to what had taken place elsewhere. After some desultory observations, Dr. Lynch's motion, somewhat modified, was passed. Dr. Granville then begged leave to propose that the Committee should make application to Her Majesty's Government for a grant to be applied to the purposes of enquiry into the important subject of the contagious or non-contagious nature of plague. This motion passed unanimously, as also one of thanks to Dr. Bowring.

Mr. Goodsir then read a paper "On the Origin and subsequent Development of the Human Teeth."

The author has observed dention commence by the formation of what he denominates the primitive dental groove, on the floor of which the rudiments of the pulps of the milk teeth appear as globular or conical papillæ; septa afterwards pass from the outer to the inner side of the groove, between the papillæ, and thus each of the latter becomes situated in an open mouthed follicle, which is the primitive condition of the future sac. After the formation of the milk follicles, the lips of the groove still remain prominent; and when in this condition he denominates it the secondary groove. The rudiments of the ten anterior permanent teeth appear as little depressions in the secondary groove, internal to the mouths of the milk follicles. The papillæ of the milk teeth now begin to be moulded into the form of pulps, a change which is synchronous with the closure of the mouths of the follicles by two or more laminæ, which agree in number, shape, and position with the cutting edges and tubercles of the future teeth. The lips and walls of the secondary groove now adhere, except in the situations of the ten depressions for the permanent teeth, and for a small extent posteriorly on each side, where a portion of the primitive dental groove remains in its original condition. In this portion the papillæ and follicle of the first large molar tooth appear, and, after it closes over, the lips of the secondary groove above it adhere, but not the walls; so that there is in this situation a cavity which produces the sacs of the two posterior permanent molars. The first large grinder may, therefore, be considered in some measure a milk tooth. The author observes, that dentition begins, and is always in advance, in the upper jaw, except in the case of the incisive teeth, which, although they appear first, are later in coming to perfection. This he explains by the tardy development of the lateral elements of the intermaxillary system. The author divides dentition into three stages. The first is one with which anatomists have hitherto been unacquainted,—viz. the follicular. The second and third they are familiar with—the saccular and the eruptive. From his researches, he concludes that the human teeth originate from mucous membrane; that the permanent teeth have no connexion with the deciduous set, and that the sac and pulps must be referred to the class of organs denominated bulbs. He anticipates the discovery of the follicular stage in the dentition of all animals, and if so, that it will explain the varying and complicated forms of the pulp and sacs.

Dr. Dalziel brought before the Section a model of an apparatus for the purpose of promoting respiration during sleep. Its object was to diminish the amount of atmospheric pressure on the surface of the body, whilst the patient was respiring atmospheric air of the usual density; and this principle might be applied in various diseased states with benefit, and particularly to the recovery of individuals labouring under suspended animation.—On the motion of Dr. Knott, it was resolved that the apparatus be recommended to the notice of the Humane Society, for the purpose of making trial of its efficacy.

A paper, entitled "Experiments and Observations on the cause of the Sounds of Respiration," by Dr. Spittal, was then read.

The object of this communication was to show, that the theory of Laennec, in regard to the cause of the respiratory sounds,—viz. that all those known by the terms vesicular, bronchial, tracheal, as well as cavernous and amphoric respiratory murmurs, are caused by the friction of the air against the parietes of the air-cells, bronchial tubes, trachea, and of cavities of different dimensions, has never been proved; and that the few experiments which have been advanced in support of it, are far from establishing the conclusions which have been deduced from them; and that it is highly probable that, according to the theory of M. Beau,* these sounds, either owe their existence to, or are in part produced or modified by, the transmission or reverberation of a sound which takes place in the superior respiratory passages, and which has been termed, by M. Beau, the "guttural" respiratory sound. In support of the first theory, it was observed, that the best and almost the only experiment was that of Magendie, in which air was blown into the lungs by means of a pair of bellows, and in which sounds, resembling the respiratory murmur, were perceived, and from which M. Magendie drew the conclusion, that because air passed to and from the lungs during this experiment, as well as during respiration, therefore, the respiratory sounds are produced by the friction of the air against the parietes of the bronchial tubes and air-cells of the lungs. It was stated that the similarity between the sound produced by a pair of

* Archives Génerales, Paris, 1834.

bellows, and the guttural sound, was admitted by Laennec; and that it was also observed that a similar sound could be produced by blowing air through almost any tube; differing in tone and degree, according to the diameter or shape of the opening in the tube—the force with which the air is made to issue from it—or the nature of the materials of which it is composed. The experiments of M. Beau, in support of his particular theory, it was noticed, were open to objections, and did not seem to bear out very clearly the conclusions at which he arrives; which may perhaps account for the neglect his view of the subject has met with. For the purpose of obviating these, and showing in a more distinct manner the probable truth of this theory, to a certain extent at least, several experiments were devised, calculated to prove this in a less objectionable manner. In these experiments no stream of air was allowed to pass through those parts the subject of observation, which were only allowed to become, and remain distended, with air; while, at the same time, the sound produced by the issuing of the air from an air-condensing apparatus, or from the mouth,—and which very nearly resembled that of the bellows, which again resembles the guttural sound, —was observed to have passed freely, in one experiment, throughout an artery of eighteen inches in length, and to be perceived very nearly, if not quite, as loud in this as in another artery connected with it, and through which a current of air passed. In another experiment, in which the lungs of a lamb were used, sounds analogous to the tracheal, bronchial, and vesicular, respiratory murmurs were distinctly perceived, although no current of air passed along the air tubes or cells; and in the case of a bladder, attached to one of the great bifurcations of the trachea, a sound louder than that in the bronchial tubes was perceived, when the former was contracted to about an inch and a half or two inches in diameter; feebler when larger; and assuming, as its size was increased, a gentle, shrill, ringing, amphoric character. In these different observations, no current of air passed along the parts the subject of examination, but was conveyed away in a manner which our space will not permit us to describe. These experiments were not advanced to prove that the guttural sound, or that which takes place in the superior respiratory passages, is the only source of the respiratory murmurs; but to show that in all probability it exerts a considerable influence, if not in producing, at least in modifying, the different respiratory sounds, known as the vesicular, bronchial, tracheal, cavernous, and amphoric respiratory murmurs, all of which have hitherto been explained according to the views of Laennec.

(Saturday, *Aug.* 25.)

The section met at half-past ten o'clock, to hear some papers, which could not be brought forward for want of time at the former sittings.

Mr. Crawford read a paper "On Anthracosis, occurring in an individual who had worked in a lead mine."

A conversation ensued as to the prevalence of this disease in the coal mines around Newcastle. The opinions seemed contradictory on the subject.

"On the Medicinal and Poisonous Properties of some of the Iodides," by Dr. A. T. Thomson.

The principal preparation whose action was detailed was the iodide of arsenic. Different modes of preparation were pointed out, its characters described, and specimens of the preparation handed round. The action of this medicine in very minute doses, from one-eighth to one-third of a grain, was peculiarly serviceable in lepra vulgaris, and chronic impetigo. A case of numerous tumours resembling carcinoma was found to yield to its continued action, and it was found equally successful in a more decided case of incipient carcinoma. Its action as a poison, when given in overdose, was minutely detailed in a series of experiments on dogs; its action being very similar to that of arsenious acid. Coloured drawings of the morbid effects on the alimentary canal were exhibited; when injected into a vein, its effect was to destroy life by destroying the irritability of the heart.

Dr. Adams read a paper "On the Placental Souffle," detailing some remarkable stethoscopic phenomena occasionally heard in connexion with it.

